# Supplementary material for: Stress and substance abuse among workers during the COVID-19 pandemic in an intensive care unit: A cross-sectional study
Source: PLoS One. 2022 Feb 10;17(2):e0263892. doi: 10.1371/journal.pone.0263892 (PMC8830709; doi:10.1371/journal.pone.0263892)
Supplement: S1 File — (DOCX) [file pone.0263892.s001.docx]

S1 File. Personal and Occupational Information (English)

Personal and Occupational Information.

| **Personal and Occupational Information** | **Choose the option that best reflects your reality.** |
| --- | --- |
| **Age** | 18-22. 23-27. 28-32. 33-37. 38-42. 43-47. 48-52. 53-57. 58-62.  63 or more. |
| **Sex** | Male. Female. Does not apply. |
| **Profession** | Physiotherapy. Medicine. Nursing. Nursing Technician. Nutrition. Psychology.  Not a health care profession. Social Services. Phonoaudiology. Pharmacist |
| **Exposition History** | So far, how many hours per week have you been in contact with environments containing infected patients during the pandemic?  I had no exposition to infected patients. Up to 24 hours.  Between 24-48 hours. Over 48 hours. |
| **Psychiatric Disease History** | Yes, I have a history of psychiatric disease.  If yes, which of the following: Mood Disorder(s). Anxiety Disorder(s). Other.    No, I don’t have a history of psychiatric disease. |
| **Previous COVID-19 Infection** | Yes.  If yes, in which patient setting have you been taken care of?  Outpatient. Inpatient. ICU.  No. |
| **Family History of COVID-19 Infection** | Yes.  If yes, in which patient setting have your relative(s) been taken care of?  Outpatient. Inpatient. ICU. Relative perished due to COVID-19.  No. |
| **Are you watching (or following online) more news than usual?** | Yes.  No. |
| **Do you live with anyone who is in one of the risk groups for COVID-19 infection? (elderly, immunosuppressed, neonate, etc)** | Yes.  No. |
| **Have you sought accommodation away from home during the pandemic?** | Yes.  No. |
| ***Do you feel that your mental health is impaired during the pandemic? (e.g., sadness, difficult to sleep, stress, anxiety, suicidal thoughts, lack of pleasure, aggressiveness)** | Yes.  No. |
