# Supplementary material for: Stress and substance abuse among workers during the COVID-19 pandemic in an intensive care unit: A cross-sectional study
Source: PLoS One. 2022 Feb 10;17(2):e0263892. doi: 10.1371/journal.pone.0263892 (PMC8830709; doi:10.1371/journal.pone.0263892)
Supplement: S2 File — (DOCX) [file pone.0263892.s002.docx]

S2 File. Personal and Professional Information (Portuguese)

Informações Pessoais e Profissionais.

| **Informações profissionais e pessoais** | **Circule a opção que mais se adequar a sua realidade.** |
| --- | --- |
| **Idade** | 18-22 23-27 28-32 33-37 38-42 43-47 48-52 53-57 58-62 63 ou mais |
| **Sexo** | Masculino Feminino Não se aplica |
| **Área de atuação** | Fisioterapia Medicina Enfermagem Auxiliar de Enfermagem Nutrição Psicologia Não profissional de saúde Serviço Social Fonoaudiologia |
| **Histórico de Exposição** | Quantas horas por semana você tem contato com setores que trabalham com doentes infectados no cenário da pandemia?  Sem exposição direta a pacientes infectados Até 24 horas  Entre 24-48 horas Acima de 48 horas |
| **Histórico de doença psiquiátrica** | Sim  Qual: Transtorno de Humor Transtorno de Ansiedade Outros    Não |
| **Histórico de infecção por COVID-19** | Sim  Qual foi o quadro clínico? Ambulatorial Internado UTI  Não |
| **Histórico de familiar infectado por COVID-19** | Sim  Qual foi o quadro clínico? Ambulatorial Internado UTI Óbito  Não |
| **Você está assistindo (ou acompanhando pela internet) a mais noticiários que o habitual?** | Sim  Não |
| **Você mora com alguém que é grupo de risco (idosos, imunossuprimido, criança, etc) para a infecção por COVID-19?** | Sim  Não |
| **Você precisou sair de casa para proteger seus familiares e evitar o contágio deles?** | Sim  Não |
| ***Você sente que sua saúde mental está pior no contexto da pandemia? (ex: tristeza, insônia, stress, ansiedade, ideação suicida, desesperança, anedonia, agressividade)** | Sim  Não |
