# Supplementary material for: Stress and substance abuse among workers during the COVID-19 pandemic in an intensive care unit: A cross-sectional study
Source: PLoS One. 2022 Feb 10;17(2):e0263892. doi: 10.1371/journal.pone.0263892 (PMC8830709; doi:10.1371/journal.pone.0263892)
Supplement: S3 File — (DOCX) [file pone.0263892.s003.docx]

S3 File. Impact of Events Scale – Revised (English)

Impact of Events Scale – Revised

INSTRUCTIONS: Below is a list of difficulties people sometimes have after stressful life

events. Please read each item, and then indicate how distressing each difficulty has been for

you during the past seven days with respect to what you have been experiencing in the pandemic. How much have you been distressed or bothered by these difficulties?

|  | **Not at all** | **A little bit** | **Moderately** | **Quite a bit** | **Extremely** |
| --- | --- | --- | --- | --- | --- |
| **1.** Any reminder brought back feelings about it. | 0 | 1 | 2 | 3 | 4 |
| **2.** I had trouble staying asleep. | 0 | 1 | 2 | 3 | 4 |
| **3.** Other things kept making me think about it. | 0 | 1 | 2 | 3 | 4 |
| **4.** I felt irritable and angry. | 0 | 1 | 2 | 3 | 4 |
| **5.** I avoided letting myself get upset when I thought about it or was reminded of it. | 0 | 1 | 2 | 3 | 4 |
| **6.** I thought about it when I didn't mean to. | 0 | 1 | 2 | 3 | 4 |
| **7.** I felt as if it hadn't happened or wasn't real. | 0 | 1 | 2 | 3 | 4 |
| **8.** I stayed away from reminders of it. | 0 | 1 | 2 | 3 | 4 |
| **9.** Pictures about it popped into my mind. | 0 | 1 | 2 | 3 | 4 |
| **10.** I was jumpy and easily startled. | 0 | 1 | 2 | 3 | 4 |
| **11.** I tried not to think about it. | 0 | 1 | 2 | 3 | 4 |
| **12.** I was aware that I still had a lot of  feelings about it, but I didn't deal with them. | 0 | 1 | 2 | 3 | 4 |
| **13.** My feelings about it were kind of numb. | 0 | 1 | 2 | 3 | 4 |
| **14.** I found myself acting or feeling like I was back at that time. | 0 | 1 | 2 | 3 | 4 |
| **15.** I had trouble falling asleep. | 0 | 1 | 2 | 3 | 4 |
| **16.** I had waves of strong feelings about it. | 0 | 1 | 2 | 3 | 4 |
| **17.** I tried to remove it from my memory. | 0 | 1 | 2 | 3 | 4 |
| **18.** I had trouble concentrating. | 0 | 1 | 2 | 3 | 4 |
| **19.** Reminders of it caused me to have  physical reactions, such as sweating, trouble breathing, nausea, or a pounding  heart. | 0 | 1 | 2 | 3 | 4 |
| **20.** I had dreams about it. | 0 | 1 | 2 | 3 | 4 |
| **21.** I felt watchful and on-guard. | 0 | 1 | 2 | 3 | 4 |
| **22.** I tried not to talk about it. | 0 | 1 | 2 | 3 | 4 |
