# Supplementary material for: Stress and substance abuse among workers during the COVID-19 pandemic in an intensive care unit: A cross-sectional study
Source: PLoS One. 2022 Feb 10;17(2):e0263892. doi: 10.1371/journal.pone.0263892 (PMC8830709; doi:10.1371/journal.pone.0263892)
Supplement: S4 File — (DOCX) [file pone.0263892.s004.docx]

S4 File. Impact of Events Scale – Revised (Portuguese)

Escala do Impacto do Evento – Revisada (IES-R) traduzida para a língua portuguesa.

Listamos abaixo as dificuldades que as pessoas algumas vezes apresentam, após passar por eventos estressantes. Com relação às memórias da pandemia, por favor, leia cada item abaixo e depois marque a coluna que melhor corresponde a seu nível de estresse, nos últimos 7 dias.

|  | **Nem um pouco** | **Um pouco** | **Moderadamente** | **Muito** | **Extremamente** |
| --- | --- | --- | --- | --- | --- |
| **1.** Qualquer lembrança trazia de volta sentimentos sobre a situação | 0 | 1 | 2 | 3 | 4 |
| **2.** Eu tinha problemas em manter o sono | 0 | 1 | 2 | 3 | 4 |
| **3.** Outros acontecimentos faziam com que eu ficasse pensando sobre a situação | 0 | 1 | 2 | 3 | 4 |
| **4.** Eu me sentia irritável e bravo | 0 | 1 | 2 | 3 | 4 |
| **5.** Eu evitava ficar chateado quando pensava sobre a situação ou era lembrado dela | 0 | 1 | 2 | 3 | 4 |
| **6.** Eu pensava sobre a situação mesmo quando não tinha intenção de pensar | 0 | 1 | 2 | 3 | 4 |
| **7.** Eu sentia como se não tivesse passado pela situação ou como se não fosse real | 0 | 1 | 2 | 3 | 4 |
| **8.** Eu me mantive longe de coisas que pudessem relembrar a situação | 0 | 1 | 2 | 3 | 4 |
| **9.** Imagens sobre a situação saltavam em minha mente | 0 | 1 | 2 | 3 | 4 |
| **10.** Eu ficava sobressaltado e facilmente alarmado | 0 | 1 | 2 | 3 | 4 |
| **11.** Eu tentei não pensar sobre a situação | 0 | 1 | 2 | 3 | 4 |
| **12.** Eu sabia que ainda tinha muitas emoções ligadas à situação, mas as evitei | 0 | 1 | 2 | 3 | 4 |
| **13.** Meus sentimentos sobre a situação estavam como que entorpecidos | 0 | 1 | 2 | 3 | 4 |
| **14.** Eu me peguei agindo ou sentindo como se estivesse de volta à situação | 0 | 1 | 2 | 3 | 4 |
| **15.** Eu tive problemas para dormir | 0 | 1 | 2 | 3 | 4 |
| **16.** Eu tive ondas de fortes emoções relativas à situação | 0 | 1 | 2 | 3 | 4 |
| **17.** Eu tentei retirar a situação da minha memória | 0 | 1 | 2 | 3 | 4 |
| **18.** Eu tive problemas de concentração | 0 | 1 | 2 | 3 | 4 |
| **19.** Lembranças da situação faziam com que eu tivesse reações físicas, como suores, problemas para respirar, náuseas ou coração disparado | 0 | 1 | 2 | 3 | 4 |
| **20.** Eu tive sonhos sobre a situação | 0 | 1 | 2 | 3 | 4 |
| **21.** Eu me sentia atento ou na defensiva | 0 | 1 | 2 | 3 | 4 |
| **22.** Eu tentei não falar sobre a situação | 0 | 1 | 2 | 3 | 4 |
