# Supplementary material for: Stress and substance abuse among workers during the COVID-19 pandemic in an intensive care unit: A cross-sectional study
Source: PLoS One. 2022 Feb 10;17(2):e0263892. doi: 10.1371/journal.pone.0263892 (PMC8830709; doi:10.1371/journal.pone.0263892)
Supplement: S5 File — (DOCX) [file pone.0263892.s005.docx]

S5 File. Alcohol, Smoking, and Substance Involvement Test (ASSIST 2.0) [English]

Alcohol, Smoking, and Substance Involvement Test (ASSIST 2.0)

| **1 – In your life, which of the following substances have you ever used?** | **No** | **Yes** |
| --- | --- | --- |
| **a. Tobacco and derivatives** | 0 | 1 |
| **b. Alcoholic drinks** | 0 | 1 |
| **c. Cannabis (marijuana, pot, grass, etc.)** | 0 | 1 |
| **d. Cocaine (coke, crack, etc.)** | 0 | 1 |
| **e. Stimulants and Methamphetamine (meth, Crystal, ecstasy, speed, etc.)** | 0 | 1 |
| **f. Inhalants (nitrous, glue, paint thinner, poppers, whippets, etc.)** | 0 | 1 |
| **g. Sedatives (sleeping pills, Valium, Xanax, tranquilizers, benzos, etc.)** | 0 | 1 |
| **h. Hallucinogens (LSD, acid, mushrooms, PCP, Special K, etc.)** | 0 | 1 |
| **i. Opioids (heroin, opium, morphine, methadone, Vicodin, etc.)** | 0 | 1 |
| **j. Any other drugs.** | 0 | 1 |

| **2 – In the past three months, how often have you used the substances you mentioned? (First drug, Second drug, etc.)** | **Never** | **Once or twice** | **Monthly** | **Weekly** | **Daily or almost daily** |
| --- | --- | --- | --- | --- | --- |
| **a. Tobacco and derivatives** | 0 | 1 | 2 | 3 | 4 |
| **b. Alcoholic drinks** | 0 | 1 | 2 | 3 | 4 |
| **c. Cannabis (marijuana, pot, grass, etc.)** | 0 | 1 | 2 | 3 | 4 |
| **d. Cocaine (coke, crack, etc.)** | 0 | 1 | 2 | 3 | 4 |
| **e. Stimulants and Methamphetamine (meth, Crystal, ecstasy, speed, etc.)** | 0 | 1 | 2 | 3 | 4 |
| **f. Inhalants (nitrous, glue, paint thinner, poppers, whippets, etc.)** | 0 | 1 | 2 | 3 | 4 |
| **g. Sedatives (sleeping pills, Valium, Xanax, tranquilizers, benzos, etc.)** | 0 | 1 | 2 | 3 | 4 |
| **h. Hallucinogens (LSD, acid, mushrooms, PCP, Special K, etc.)** | 0 | 1 | 2 | 3 | 4 |
| **i. Opioids (heroin, opium, morphine, methadone, Vicodin, etc.)** | 0 | 1 | 2 | 3 | 4 |
| **j. Any other drugs.** | 0 | 1 | 2 | 3 | 4 |

| **3 – During the past three months, how often have you had a strong desire or urge to use it? (First drug, Second drug, etc.)** | **Never** | **Once or twice** | **Monthly** | **Weekly** | **Daily or almost daily** |
| --- | --- | --- | --- | --- | --- |
| **a. Tobacco and derivatives** | 0 | 1 | 2 | 3 | 4 |
| **b. Alcoholic drinks** | 0 | 1 | 2 | 3 | 4 |
| **c. Cannabis (marijuana, pot, grass, etc.)** | 0 | 1 | 2 | 3 | 4 |
| **d. Cocaine (coke, crack, etc.)** | 0 | 1 | 2 | 3 | 4 |
| **e. Stimulants and Methamphetamine (meth, Crystal, ecstasy, speed, etc.)** | 0 | 1 | 2 | 3 | 4 |
| **f. Inhalants (nitrous, glue, paint thinner, poppers, whippets, etc.)** | 0 | 1 | 2 | 3 | 4 |
| **g. Sedatives (sleeping pills, Valium, Xanax, tranquilizers, benzos, etc.)** | 0 | 1 | 2 | 3 | 4 |
| **h. Hallucinogens (LSD, acid, mushrooms, PCP, Special K, etc.)** | 0 | 1 | 2 | 3 | 4 |
| **i. Opioids (heroin, opium, morphine, methadone, Vicodin, etc.)** | 0 | 1 | 2 | 3 | 4 |
| **j. Any other drugs.** | 0 | 1 | 2 | 3 | 4 |

| **4 – During the past three months, how often has your use of it led to health, social, legal, or financial problems? (First drug, Second drug, etc.)** | **Never** | **Once or twice** | **Monthly** | **Weekly** | **Daily or almost daily** |
| --- | --- | --- | --- | --- | --- |
| **a. Tobacco and derivatives** | 0 | 1 | 2 | 3 | 4 |
| **b. Alcoholic drinks** | 0 | 1 | 2 | 3 | 4 |
| **c. Cannabis (marijuana, pot, grass, etc.)** | 0 | 1 | 2 | 3 | 4 |
| **d. Cocaine (coke, crack, etc.)** | 0 | 1 | 2 | 3 | 4 |
| **e. Stimulants and Methamphetamine (meth, Crystal, ecstasy, speed, etc.)** | 0 | 1 | 2 | 3 | 4 |
| **f. Inhalants (nitrous, glue, paint thinner, poppers, whippets, etc.)** | 0 | 1 | 2 | 3 | 4 |
| **g. Sedatives (sleeping pills, Valium, Xanax, tranquilizers, benzos, etc.)** | 0 | 1 | 2 | 3 | 4 |
| **h. Hallucinogens (LSD, acid, mushrooms, PCP, Special K, etc.)** | 0 | 1 | 2 | 3 | 4 |
| **i. Opioids (heroin, opium, morphine, methadone, Vicodin, etc.)** | 0 | 1 | 2 | 3 | 4 |
| **j. Any other drugs.** | 0 | 1 | 2 | 3 | 4 |

| **5 – During the past three months, how often have you failed to do what was normally expected of you because of your use of it? (First drug, Second drug, etc.)** | **Never** | **Once or twice** | **Monthly** | **Weekly** | **Daily or almost daily** |
| --- | --- | --- | --- | --- | --- |
| **a. Tobacco and derivatives** | 0 | 1 | 2 | 3 | 4 |
| **b. Alcoholic drinks** | 0 | 1 | 2 | 3 | 4 |
| **c. Cannabis (marijuana, pot, grass, etc.)** | 0 | 1 | 2 | 3 | 4 |
| **d. Cocaine (coke, crack, etc.)** | 0 | 1 | 2 | 3 | 4 |
| **e. Stimulants and Methamphetamine (meth, Crystal, ecstasy, speed, etc.)** | 0 | 1 | 2 | 3 | 4 |
| **f. Inhalants (nitrous, glue, paint thinner, poppers, whippets, etc.)** | 0 | 1 | 2 | 3 | 4 |
| **g. Sedatives (sleeping pills, Valium, Xanax, tranquilizers, benzos, etc.)** | 0 | 1 | 2 | 3 | 4 |
| **h. Hallucinogens (LSD, acid, mushrooms, PCP, Special K, etc.)** | 0 | 1 | 2 | 3 | 4 |
| **i. Opioids (heroin, opium, morphine, methadone, Vicodin, etc.)** | 0 | 1 | 2 | 3 | 4 |
| **j. Any other drugs.** | 0 | 1 | 2 | 3 | 4 |

| **6 – Has a friend or relative or anyone else ever expressed concern about your use of it? (First drug, Second drug, etc.)** | **No, never** | **Yes, in the past 3 months** | **Yes, but not in the past 3 months** |
| --- | --- | --- | --- |
| **a. Tobacco and derivatives** | 0 | 1 | 2 |
| **b. Alcoholic drinks** | 0 | 1 | 2 |
| **c. Cannabis (marijuana, pot, grass, etc.)** | 0 | 1 | 2 |
| **d. Cocaine (coke, crack, etc.)** | 0 | 1 | 2 |
| **e. Stimulants and Methamphetamine (meth, Crystal, ecstasy, speed, etc.)** | 0 | 1 | 2 |
| **f. Inhalants (nitrous, glue, paint thinner, poppers, whippets, etc.)** | 0 | 1 | 2 |
| **g. Sedatives (sleeping pills, Valium, Xanax, tranquilizers, benzos, etc.)** | 0 | 1 | 2 |
| **h. Hallucinogens (LSD, acid, mushrooms, PCP, Special K, etc.)** | 0 | 1 | 2 |
| **i. Opioids (heroin, opium, morphine, methadone, Vicodin, etc.)** | 0 | 1 | 2 |
| **j. Any other drugs.** | 0 | 1 | 2 |

| **7 – Have you ever tried and failed to control, cut down or stop using it? (First drug, Second drug, etc.)** | **No, never** | **Yes, in the past 3 months** | **Yes, but not in the past 3 months** |
| --- | --- | --- | --- |
| **a. Tobacco and derivatives** | 0 | 1 | 2 |
| **b. Alcoholic drinks** | 0 | 1 | 2 |
| **c. Cannabis (marijuana, pot, grass, etc.)** | 0 | 1 | 2 |
| **d. Cocaine (coke, crack, etc.)** | 0 | 1 | 2 |
| **e. Stimulants and Methamphetamine (meth, Crystal, ecstasy, speed, etc.)** | 0 | 1 | 2 |
| **f. Inhalants (nitrous, glue, paint thinner, poppers, whippets, etc.)** | 0 | 1 | 2 |
| **g. Sedatives (sleeping pills, Valium, Xanax, tranquilizers, benzos, etc.)** | 0 | 1 | 2 |
| **h. Hallucinogens (LSD, acid, mushrooms, PCP, Special K, etc.)** | 0 | 1 | 2 |
| **i. Opioids (heroin, opium, morphine, methadone, Vicodin, etc.)** | 0 | 1 | 2 |
| **j. Any other drugs.** | 0 | 1 | 2 |

| **8 – Have you ever used any drug by injection? (NON-MEDICAL USE ONLY)** | **No, never** | **Yes, in the past 3 months** | **Yes, but not in the past 3 months** |
| --- | --- | --- | --- |
|  | 0 | 1 | 2 |
