# Supplementary material for: Stress and substance abuse among workers during the COVID-19 pandemic in an intensive care unit: A cross-sectional study
Source: PLoS One. 2022 Feb 10;17(2):e0263892. doi: 10.1371/journal.pone.0263892 (PMC8830709; doi:10.1371/journal.pone.0263892)
Supplement: S6 File — (DOCX) [file pone.0263892.s006.docx]

S6 File. Alcohol, Smoking, and Substance Involvement Test (ASSIST 2.0) [Portuguese]

Questionário para triagem do uso de álcool, tabaco a outras substancias (ASSIST 2.0)

| **1 – Na sua vida, qual (is) dessas substâncias você já usou? (SOMENTE USO NÃO-MÉDICO)** | **NÃO** | **SIM** |
| --- | --- | --- |
| a. Derivados do tabaco (cigarros, charuto, cachimbo, fumo de corda...) | 0 | 1 |
| b. Bebidas alcoólicas (cerveja, vinho, destilados como pinga, uísque, vodka, vermutes...) | 0 | 1 |
| c. Maconha (baseado, erva, haxixe...) | 0 | 1 |
| d. Cocaína, crack (pó, pedra, branquinha, nuvem...) | 0 | 1 |
| e. Estimulantes como anfetaminas ou ecstasy (bolinhas, rebites...) | 0 | 1 |
| f. Inalantes (cola de sapateiro, cheirinho-da-loló, tinta, gasolina, éter, lança-perfume,benzina...) | 0 | 1 |
| g. Hipnóticos/sedativos (remédios para dormir: diazepam, lorazepan, lorax, dienpax, rohypnol). | 0 | 1 |
| h. Drogas alucinógenas (como LSD, ácido, chá-de-lírio, cogumelos...) | 0 | 1 |
| i. Opióides (heroína, morfina, metadona, codeína...) | 0 | 1 |
| j. Outras, Especificar: | 0 | 1 |

| **2 – Durante os três últimos meses, com que freqüência você utilizou essa(s) substância(s) que mencionou? (Primeira droga, depois a segunda droga, etc)** | **Nunca** | **1 ou 2 vezes** | **Mensalmente** | **Semanalmente** | **Diariamente ou quase todo dia** |
| --- | --- | --- | --- | --- | --- |
| a. Derivados do tabaco (cigarros, charuto, cachimbo, fumo de corda...) | 0 | 1 | 2 | 3 | 4 |
| b. Bebidas alcoólicas (cerveja, vinho, destilados como pinga, uísque, vodka, vermutes...) | 0 | 1 | 2 | 3 | 4 |
| c. Maconha (baseado, erva, haxixe...) | 0 | 1 | 2 | 3 | 4 |
| d. Cocaína, crack (pó, pedra, branquinha, nuvem...) | 0 | 1 | 2 | 3 | 4 |
| e. Estimulantes como anfetaminas ou ecstasy (bolinhas, rebites...) | 0 | 1 | 2 | 3 | 4 |
| f. Inalantes (cola de sapateiro, cheirinho-da-loló, tinta, gasolina, éter, lança-perfume,benzina...) | 0 | 1 | 2 | 3 | 4 |
| g. Hipnóticos/sedativos (remédios para dormir: diazepam, lorazepan, lorax, dienpax, rohypnol). | 0 | 1 | 2 | 3 | 4 |
| h. Drogas alucinógenas (como LSD, ácido, chá-de-lírio, cogumelos...) | 0 | 1 | 2 | 3 | 4 |
| i. Opióides (heroína, morfina, metadona, codeína...) | 0 | 1 | 2 | 3 | 4 |
| j. Outras, Especificar: | 0 | 1 | 2 | 3 | 4 |

| **3 – Durante os três últimos meses, com que freqüência você teve um forte desejo ou urgência em consumir? (Primeira droga, depois a segunda droga, etc)** | **Nunca** | **1 ou 2 vezes** | **Mensalmente** | **Semanalmente** | **Diariamente ou quase todo dia** |
| --- | --- | --- | --- | --- | --- |
| a. Derivados do tabaco (cigarros, charuto, cachimbo, fumo de corda...) | 0 | 1 | 2 | 3 | 4 |
| b. Bebidas alcoólicas (cerveja, vinho, destilados como pinga, uísque, vodka, vermutes...) | 0 | 1 | 2 | 3 | 4 |
| c. Maconha (baseado, erva, haxixe...) | 0 | 1 | 2 | 3 | 4 |
| d. Cocaína, crack (pó, pedra, branquinha, nuvem...) | 0 | 1 | 2 | 3 | 4 |
| e. Estimulantes como anfetaminas ou ecstasy (bolinhas, rebites...) | 0 | 1 | 2 | 3 | 4 |
| f. Inalantes (cola de sapateiro, cheirinho-da-loló, tinta, gasolina, éter, lança-perfume,benzina...) | 0 | 1 | 2 | 3 | 4 |
| g. Hipnóticos/sedativos (remédios para dormir: diazepam, lorazepan, lorax, dienpax, rohypnol). | 0 | 1 | 2 | 3 | 4 |
| h. Drogas alucinógenas (como LSD, ácido, chá-de-lírio, cogumelos...) | 0 | 1 | 2 | 3 | 4 |
| i. Opióides (heroína, morfina, metadona, codeína...) | 0 | 1 | 2 | 3 | 4 |
| j. Outras, Especificar: | 0 | 1 | 2 | 3 | 4 |

| **4 – Durante os três últimos meses, com que freqüência o seu consumo de (Primeira droga, depois a segunda droga, etc) resultou em problema de saúde, social, legal ou financeiro?** | **Nunca** | **1 ou 2 vezes** | **Mensalmente** | **Semanalmente** | **Diariamente ou quase todo dia** |
| --- | --- | --- | --- | --- | --- |
| a. Derivados do tabaco (cigarros, charuto, cachimbo, fumo de corda...) | 0 | 1 | 2 | 3 | 4 |
| b. Bebidas alcoólicas (cerveja, vinho, destilados como pinga, uísque, vodka, vermutes...) | 0 | 1 | 2 | 3 | 4 |
| c. Maconha (baseado, erva, haxixe...) | 0 | 1 | 2 | 3 | 4 |
| d. Cocaína, crack (pó, pedra, branquinha, nuvem...) | 0 | 1 | 2 | 3 | 4 |
| e. Estimulantes como anfetaminas ou ecstasy (bolinhas, rebites...) | 0 | 1 | 2 | 3 | 4 |
| f. Inalantes (cola de sapateiro, cheirinho-da-loló, tinta, gasolina, éter, lança-perfume,benzina...) | 0 | 1 | 2 | 3 | 4 |
| g. Hipnóticos/sedativos (remédios para dormir: diazepam, lorazepan, lorax, dienpax, rohypnol). | 0 | 1 | 2 | 3 | 4 |
| h. Drogas alucinógenas (como LSD, ácido, chá-de-lírio, cogumelos...) | 0 | 1 | 2 | 3 | 4 |
| i. Opióides (heroína, morfina, metadona, codeína...) | 0 | 1 | 2 | 3 | 4 |
| j. Outras, Especificar: | 0 | 1 | 2 | 3 | 4 |

| **5 – Durante os três últimos meses, com que freqüência por causa do seu uso de (Primeira droga, depois a segunda droga, etc) você deixou de fazer coisas que eram normalmente esperadas por você?** | **Nunca** | **1 ou 2 vezes** | **Mensalmente** | **Semanalmente** | **Diariamente ou quase todo dia** |
| --- | --- | --- | --- | --- | --- |
| a. Derivados do tabaco (cigarros, charuto, cachimbo, fumo de corda...) | 0 | 1 | 2 | 3 | 4 |
| b. Bebidas alcoólicas (cerveja, vinho, destilados como pinga, uísque, vodka, vermutes...) | 0 | 1 | 2 | 3 | 4 |
| c. Maconha (baseado, erva, haxixe...) | 0 | 1 | 2 | 3 | 4 |
| d. Cocaína, crack (pó, pedra, branquinha, nuvem...) | 0 | 1 | 2 | 3 | 4 |
| e. Estimulantes como anfetaminas ou ecstasy (bolinhas, rebites...) | 0 | 1 | 2 | 3 | 4 |
| f. Inalantes (cola de sapateiro, cheirinho-da-loló, tinta, gasolina, éter, lança-perfume,benzina...) | 0 | 1 | 2 | 3 | 4 |
| g. Hipnóticos/sedativos (remédios para dormir: diazepam, lorazepan, lorax, dienpax, rohypnol). | 0 | 1 | 2 | 3 | 4 |
| h. Drogas alucinógenas (como LSD, ácido, chá-de-lírio, cogumelos...) | 0 | 1 | 2 | 3 | 4 |
| i. Opióides (heroína, morfina, metadona, codeína...) | 0 | 1 | 2 | 3 | 4 |
| j. Outras, Especificar: | 0 | 1 | 2 | 3 | 4 |

| **6 – Há amigos, parentes ou outra pessoa que tenha demonstrado preocupação com seu uso de (Primeira droga, depois a segunda droga, etc)?** | **NÃO, nunca** | **SIM, mas não nos últimos 3 meses** | **SIM, nos últimos 3 meses** |
| --- | --- | --- | --- |
| a. Derivados do tabaco (cigarros, charuto, cachimbo, fumo de corda...) | 0 | 1 | 2 |
| b. Bebidas alcoólicas (cerveja, vinho, destilados como pinga, uísque, vodka, vermutes...) | 0 | 1 | 2 |
| c. Maconha (baseado, erva, haxixe...) | 0 | 1 | 2 |
| d. Cocaína, crack (pó, pedra, branquinha, nuvem...) | 0 | 1 | 2 |
| e. Estimulantes como anfetaminas ou ecstasy (bolinhas, rebites...) | 0 | 1 | 2 |
| f. Inalantes (cola de sapateiro, cheirinho-da-loló, tinta, gasolina, éter, lança-perfume,benzina...) | 0 | 1 | 2 |
| g. Hipnóticos/sedativos (remédios para dormir: diazepam, lorazepan, lorax, dienpax, rohypnol). | 0 | 1 | 2 |
| h. Drogas alucinógenas (como LSD, ácido, chá-de-lírio, cogumelos...) | 0 | 1 | 2 |
| i. Opióides (heroína, morfina, metadona, codeína...) | 0 | 1 | 2 |
| j. Outras, Especificar: | 0 | 1 | 2 |

| **7 – Alguma vez você já tentou controlar, diminuir ou parar o uso de (Primeira droga, depois a segunda droga, etc)?** | **NÃO, nunca** | **SIM, mas não nos últimos 3 meses** | **SIM, nos últimos 3 meses** |
| --- | --- | --- | --- |
| a. Derivados do tabaco (cigarros, charuto, cachimbo, fumo de corda...) | 0 | 1 | 2 |
| b. Bebidas alcoólicas (cerveja, vinho, destilados como pinga, uísque, vodka, vermutes...) | 0 | 1 | 2 |
| c. Maconha (baseado, erva, haxixe...) | 0 | 1 | 2 |
| d. Cocaína, crack (pó, pedra, branquinha, nuvem...) | 0 | 1 | 2 |
| e. Estimulantes como anfetaminas ou ecstasy (bolinhas, rebites...) | 0 | 1 | 2 |
| f. Inalantes (cola de sapateiro, cheirinho-da-loló, tinta, gasolina, éter, lança-perfume,benzina...) | 0 | 1 | 2 |
| g. Hipnóticos/sedativos (remédios para dormir: diazepam, lorazepan, lorax, dienpax, rohypnol). | 0 | 1 | 2 |
| h. Drogas alucinógenas (como LSD, ácido, chá-de-lírio, cogumelos...) | 0 | 1 | 2 |
| i. Opióides (heroína, morfina, metadona, codeína...) | 0 | 1 | 2 |
| j. Outras, Especificar: | 0 | 1 | 2 |

| **8 – Alguma vez você já usou drogas por injeção? (Apenas uso não-médico)** | **NÃO, nunca** | **SIM, mas não nos últimos 3 meses** | **SIM, nos últimos 3 meses** |
| --- | --- | --- | --- |
|  | 0 | 1 | 2 |
