# Supplementary material for: Stress and substance abuse among workers during the COVID-19 pandemic in an intensive care unit: A cross-sectional study
Source: PLoS One. 2022 Feb 10;17(2):e0263892. doi: 10.1371/journal.pone.0263892 (PMC8830709; doi:10.1371/journal.pone.0263892)
Supplement: Attachment — Submitted filename: Response to Reviewers.docx [file pone.0263892.s010.docx]

**Response to the Editor and Reviewers**

Dear Reviewers and Dr. Patel (Editorial Board of PLOS One). We are pleased to share with you our response to the comments made regarding our manuscript entitled “Stress and Substance Abuse among workers during the COVID-19 Pandemic in an Intensive Care Unit: a cross-sectional study”. We hope that our replies can properly address the issues highlighted by you. Thank you for your attention to this.

Sincerely,

Diego V. S. Pestana (on behalf of the authors)

**Comments – Editor:**

**1. Please review your reference list to ensure that it is complete and correct. If you have cited papers that have been retracted, please include the rationale for doing so in the manuscript text, or remove these references and replace them with relevant current references. Any changes to the reference list should be mentioned in the rebuttal letter that accompanies your revised manuscript. If you need to cite a retracted article, indicate the article’s retracted status in the References list and also include a citation and full reference for the retraction notice.**

Author’s Reply: Dear Dr. Patel, thank you for your help with this manuscript. We reviewed the reference list completely and, although we could not find any retracted paper, we identified some points that could be optimized, as discussed below (between brackets we informed which action was made regarding each reference). Additionally, we checked all the references to make sure they were available online at their original publisher/journal website, without retraction notes. All references were written according to the Vancouver style, as indicated at PLOS One website.

**Reference #7**: Baldisseri MR. Impaired healthcare professional. Critical Care Medicine. 2007 Feb;35(Suppl):S106–16.

[Substitution]

Although the article appears after a simple search on PubMed, whenever we tried to access it using the publisher/journal website the link led us to a blank webpage. We searched for retraction notes or such type of publication regarding this paper but there was none. Therefore, we decided to look for a new reference article to substitute it. After reading several other papers, we chose the article below, which also supports what we wrote in the respective excerpt of the presenting manuscript.

*“DeFord S, Bonom J, Durbin T. A review of literature on substance abuse among anaesthesia providers. Journal of Research in Nursing. 2019 Dec 22;24(8):587–600.”*

**Reference #15**: Group WAW. The Alcohol, Smoking and Substance Involvement Screening Test (ASSIST): development, reliability and feasibility. Addiction. 2002 Sep;97(9):1183–94.

[Substitution]

Although the previous reference also contained information regarding the ASSIST 2.0 tool, interpreting scores based solely on this paper could be difficult for readers. The new reference refers to the manual more recently published on behalf of the World Health Organization and allows readers to quickly find the information to which our study refers. Therefore, we thought it should substitute the former reference.

*“Humeniuk R, Ali R, Babor TF, Farrell M, Formigoni ML, Jittiwutikarn J, et al. Validation of the alcohol, smoking and substance involvement screening test (ASSIST). Addiction. 2008 Jun;103(6):1039–47.”*

**Reference #17**: Weiss DS. The Impact of Event Scale: Revised. In: Wilson JP, Tang CS, editors. Cross-cultural assessment of psychological trauma and PTSD  . New York: Springer; 2007. p. 219–38.

[Correction]

The first citation was incorrect. It improperly mixed information regarding the book and the chapter being cited. We corrected it with the proper citation, as listed below.

*“Weiss DS. The Impact of Event Scale: Revised. In: Cross-Cultural Assessment of Psychological Trauma and PTSD. Boston, MA: Springer US; p. 219–38.”*

**Comments - Reviewer #1**

**I thank the authors for careful consideration of my previous comments. I am largely satisfied with the revisions made, but still have a few areas of concern as highlighted below:**

**1. In the Results section, Paragraph on "Psychiatric Medical History", I believe the p-values reported are erroneous. It should be p = .034 and p = .044. Similarly, the p-values in Table 3.2 will need to be checked again to ensure that they correspond to the text.**

Author’s Reply: Dear Reviewer #1, thank you for your very constructive comments. After carefully reviewing the paragraph you mentioned, we identified a discordance between the p values presented in the paragraph and in Table 3.1. **We reassessed our statistical tests and the correct values are p = 0.341 (history of psychiatric disease) and p = 0.44 (history of anxiety disorders).** The values were properly corrected (please find our STATA output below, with the respective p values). Also, we corrected the tables’ titles according to the new order they were cited in the text. Former Table 3.1 was renamed to Table 3.2; former Table 3.2 was renamed to Table 3.1.

In this paragraph we highlighted some factors that had exhibited what we considered an association trend towards higher IES-R scores. Although they provided some interesting insights regarding the issues involved with stress and substance abuse among participants, those factors did not achieve statistical significance when considering p <0.05. Therefore, we believe that the terms used to describe those findings should be substituted: we chose “Factors without an association with IES-R scores”. Although the association between those factors and IES-R scores did not achieve statistical significance, the presenting results may still be interesting to readers, and future studies with more statistical power may show different results. Finally, we chose to present those elements in a different table (i.e., Table 3.1) to make sure readers would not confuse them with the ones that achieved statistical significance (i.e., Table 3.2).

Finally, we merged the subtopics “Personal factors” and “Occupational factors”, creating a new subtopic entitled “Personal and occupational factors”. We believe that this change made the excerpt conciser to eventual readers.

We hope your concerns regarding this topic were properly and satisfactorily addressed. Thank you for your valuable suggestions!

**2. There are still issues with the language in multiple areas, although I greatly appreciate the effort undertaken by the authors to improve the language of the manuscript thus far.**

Author’s Reply: Again, we are pleased to fully accept your suggestions regarding English issues. Below you will find a list with corrections made after your suggestions, following the same order you presented them. We hope those changes shall address your concerns regarding this matter.

**- [Incomplete sentence] "All the professionals working at an oncological COVID-19 ICU, regardless of their role (health professionals and non-health professionals), who were on duty for at least during one shift through the period of July to October/2020."**

Author’s Reply: we added the excerpt “were invited to participate in this study” after the sentence mentioned.

**- [Error indicated in caps] "Professionals with infected relatives .... significantly more likely to score higher in the IES-R, therefore more likely to suffer WITH PTSD or ASD."**

Author’s Reply: we substituted the word “with” by “from” (i.e., to suffer from PTSD or ASD).

**- [Error indicated in caps] "There was an association trend between higher scores and those who had sought alternative accommodation AWAY FROM during the pandemic ...."**

Author’s Reply: we completed the sentence with the word “home” (i.e., away from home during the pandemic). To ensure this error wasn’t present in other similar sentences, we checked the other parts in which this term was used, and they were properly written.

**- [Error indicated in caps] "For cannabis, stimulants, and cocaine, higher scores had an ... direct EXPOSITION to infected patients."**

Author’s Reply: we substituted the word “exposition” by “exposure”. Like what we did in the previous suggestion, all other similar excerpts were checked for mistakes, and they were properly written.

**- [Error indicated in caps] "It is possible that those results REFLEX the availability of those substances."**

Author’s Reply: we substituted the word “reflex” by “reflected”. No other similar mistakes were found.

**- ["Provided that" should be replaced with "GIVEN that some of those workers MAY HAVE HAD.."] Provided that some of those workers had psychological or psychiatric reasons for work leave, our findings can be underestimated.**

Author’s Reply: we replaced the terms as mentioned above.

**- [Should be phrased as "study had no FOLLOW-UP sessions] "Fourthly, as a cross-sectional study, it was not possible to determine causal relationships nor risk factors and the participants were not followed."**

Author’s Reply: we rephrased this excerpt as “Fourthly, this study had no follow-up sessions. Therefore, it was not possible to determine causal relationships or risk factors”.

**3. Minor suggestion to change the header "ASSIST 2.0 Results" to "Associations with substance abuse" instead.**

Author’s Reply: we changed the header as suggested; we agree that the suggested version is more suitable to readers. Thank you!

**4. Please provide the citation for the cut-off points/categorization of IES and ASSIST 2.0 scores after listing the cut-offs in order to allow readers to easily search for the original paper that provides these cut-offs.
i.e "The IES-R scores were categorized as follows: 1-11 = few/no signs of ASD/PTSD; 12-32 = several signs of ASD/PTSD, patient monitoring is recommended; ≥33 = highly suggestive of ASD/PTSD, immediate psychiatric evaluation is recommended [INSERT CITATION HERE]."**

Author’s Reply: we inserted the respective citations to the IES-R and ASSIST 2.0 scores whenever those categorizations were mentioned.

**5. Authors should consider elaborating a little on how their findings may be generalizable to ICUs outside Brazil. A suggestion is given below.
"the current results are in accordance those presented by other authors, in other populations of ICU workers (e.g., Netherlands [4], Canada [20], United States [21], and England [19]), suggesting that findings from this study are applicable and should be taken into consideration by professionals working in ICUs globally."**

Author’s Reply: after reading your suggestion, we added the excerpt “Thus, it is likely that the findings of the presenting study could be generalizable to ICU workers from different populations and should be taken into consideration by professionals working globally”. We agree that such a phrase may clarify the main idea of this paragraph. Thank you!

**Comments - Reviewer #2**

**The authors have responded in a satisfactory manner to all comments. I have no additional comments to make.**

Author’s Reply: We appreciate your attention when reviewing this manuscript. We hope the corrections made after your previous comments had satisfactorily addressed your concerns and improved the quality of the current manuscript.

**Comments - Reviewer #3**

**The design of the study is appropriate. The writing is in a good standard of English, grammatically correct and easy to understand. The results are clearly presented and explained.**

Author’s Reply: We are glad to read your comments to this manuscript, thank you for your help during this review process.
